# 

**Published:** 1866-08

**Authors:** 


### INDEX.

Page.

**A.**

A Diploma ........ 191
A Paper on "Surgical Remedies," Vesicants, Rubifacients, Setons, Issues, etc ........ 281
Abbott, George, M. D. History of Diphtheria, in the town of Hamburg and vicinity ........ 241
Abbott, George, M. D. Letter from ........ 303
Abbott, Samuel L. Translation of a Report to the International Sanitary Conference, on the Origin, Endemnicity, Transmissibility and Propagation of Asiatic Cholera ........
Abstract of Proceedings of the Buffalo Medical Association ........ 49
83, 133, 179, 214, 281, 354, 369, 411, 451.
Abortion, Criminal, and the Public Press .. 358
Action of Bromide of Potassium ........ 411
Acton, William, M. P. C. S. Functions and Disorders of the Reproductive Organs, in Childhood, Youth, Adult Age and Advanced Life ........ 319
Acute Pneumonia, Treatment of ........ 28
Acute Rheumatism, Trousseau's Syrup of Lime, in the Treatment of ........ 440
Advertisement, Physician's ........ 95
Aitkin, William, M. D. Science and Practice of Medicine, vol. 1 ........ 238
Albumen of the Blood in Cholera ........ 72
Albumenuria, Nervous, Idiopathic, and Albumenuria of diseased Kidneys, mode of distinguishing between ........ 27
Allopathist, Letter from a converted ........ 15
Alum, Ammonio Ferric, in the Treatment of Hydrocele. By H. R Rogers, M. D. 418
American Medical Association ........ 321
American Medical Association, Eighteenth Annual Meeting of ........ 420
American Naturalist ........ 368, 449
American Physicians Deceased, Biographical Dictionary of ........ 322
Amputation, Pyæmia after. By E. R. Barnes, M. D. ........ 385
Anaesthetic, Local ........ 30
Anaesthetic, New ........ 103
Anaesthetic, Protoxyde of Azote as an Agent, (Translated from the French.) By C. C. F. Gay, M. D. ........ 43
Animals, Cruelty to ........ 317, 367
Animal Economy—on the Mutual Action of the Elements of Soluble Salts, without and within ........ 139
Annual Meeting of the Eighteenth American Medical Association ........ 449
Annual Meeting of the Erie County Medical Society ........ 320
Annual Meeting of the State Medical Association ........ 235
Anstie, Francis Edmund, M. D., F. R. C. P. Notes on Epidemics ........ 239
Antiseptic, New Form for Local use ........ 442
Aphasia, a case of. By Jos. G. Richardson, M. D. ........ 211
Appearance of Man on our Planet ........ 368
Article upon "Cruelty to Animals," ........ 367
Association, American Medical ........ 321
Association, American Medical, Eighteenth Annual Meeting of ........ 420
Association, Buffalo Medical, Proceedings of, 49, 83, 133, 179, 214, 281, 354, 369, 411, 451.

Page.

Ashurst, John, Jr., A. M., M. D. Injuries of the Spine ........ 363
Atlantic Monthly ........ 280
Austria Joining the Society for the Relief of Suffering on the Battle-Field ........ 82
Azote, Protoxyde of, as an Anaesthetic Agent. (Translated from the French,) By C. C. F. Gay, M. D. ........ 43

**B.**

Baker, George F., D. D. S. Nitrous Oxide .. 112
Barnes, E. R., M. D. Pyaemia after Amputation ........ 385
Bartholow, Robert, M. D. On Spermatorrhœa ........ 79
Baxter, J. H., M.D., Col. U. S. Vols. Report to the War Department upon the Prevailing Diseases in the United States .. 237
Bedford, Prof. Gunning S., Medical Works in Foreign Countries ........ 192
Bennett, Henry, M. D., On the Treatment of Consumption by Hygiene, Climate and Medicine ........ 217, 307
Biographical Dictionary of Deceased American Physicians ........ 322
Black Death, "Febris Nigra," ........ 28
Blood, Albumen of, in Cholera ........ 72
Books and Pamphlets Received. 39, 80, 113, 156, 197, 240, 319, 365, 407, 449, 484.
Bowman, John E., M. D., F. C. S., Practical Chemistry ........ 239
Bromide of Potassium, Action of ........ 441
Bronchocele, removal of ........ 35
Buck, Gurdon S. M. D., Improved Extension apparatus in Fracture of the Thigh 356
Buffalo General Hospital, Extract from the Report of the Superintendent ........ 231
Buffalo Health, Epidemic Disease ........ 33
Buffalo Medical Association, 49, 83, 133, 179, 214, 281, 354, 369, 411, 451.
Buffalo Medical College, class in ........ 113
Buffalo Medical College, Commencement Exercises ........ 272
Burrall F. S., M. D. Asiatic Cholera ........ 38

**C.**

Canniff, William, M D., M. R. C. S. A Manual of the Principles of Surgery ........ 194
Carbolic Acid, Condy's ........ 186
Case of Constipation, with a subsequent Discharge of living Maggots. By A. Janson, M. D. ........ 281
Case of forcible tearing away of the Uterus 32
Case of Menstruation in a child five years of age ........ 66
Chambers, Thomas King, M D., Diseases of the Digestive Organs functionally treated ........ 405
Chambers, Thomas King, M. D. Renewal of Life ........ 318
Charge to the Graduating class. By Prof. James P. White ........ 328
Cheever, David W., M. D. Œsophogotomy for the Removal of foreign Bodies ........ 406
Chloroform, Death from, in Toronto, C. W. 323
Chloroform, Internal use of in the Treatment of Delirium Tremens ........ 145
Cholera, Albumen of the Blood in ........ 72
Cholera, Cause of ........ 442
Cholera, Conference at Constantinople, Report of ........ 17

Page.
Cholera, Facts connected with the recent spread of.... 54
Cholera, Hypodermic Injections of warm water in.... 143
Cholera, Probable Appearance, Cause and Prevention.... 443
Cholera, Remarks on. By Thos. F. Rochester, M. D. (continued).... 1
Citrate of Soda in Diabetes.... 106
Clark, S. F., M. D. Poisoning by Strychnia 135
Clark, S.F., M. D. Extra Uterine Pregnancy 135
Clitorodectomy.... 368
Clinical Remarks upon Surgical cases in the Buffalo General Hospital. By J. F. Miner, M. D.... 265
Commission, Sanitary, Contributions for Medical Volume, to be published by the 105
Commencement Exercises of the Buffalo Medical College.... 274
Completion of Vol. 1.... 478
Compressed Air Cure.... 194
Condy's Fluid and Carbolic Acid.... 186
Congenital Strangulated Hernia, Case of. By John Root, M. D.... 474
Consumption, Treatment of, by Hygiene, Climate and Medicine. By Henry Bennett, M. D....217, 307
Contributions for the Medical Volume to be published under the direction of the Sanitary Commission.... 105
Convention, Medical.... 320
Convention, Medical Teachers.... 404
Corrections by the Northwestern Christian Advocate.... 390
Cornell, Wm., M. D., LL. D. Letter from.. 14
Cornell, Wm., M. D., LL. D. The Beacon.. 79
Criminal Abortion and the Public Press... 358
Cruelty to Animals.... 317
Cruelty to Animals, Article.... 367

**D.**

Da Costa, J. M., M. D. Medical Diagnosis, 110
Da Costa, J. M., M. D. Inhalations in the Treatment of Diseases of the Respiratory Organs.... 407
Davis, Charles H. S., M.D., Letter from.... 396
Davis, Charles H. S., M.D., Nasal Polypus. 259
Death from Chloroform in Toronto, C. W... 323
Delirium Tremens. Internal use of Chloroform in the treatment of.... 45
Derby, Hasket, M. D. (Translation from the German.) Clinical Lectures on Amblyopia and Amaurosis and Extraction of Cataract, by Prof. A. Von Graefe.... 238
Diabetes, Citrate of Soda in.... 106
Dictionary, Biographical of Deceased American Physicians.... 322
Diphtheria, History of, in the town of Hamburg and vicinity. By Geo. Abbott, M.D. 241
Diphtheria, treatment of, with Hyposulphite of Soda.... 73
Discription of an Improved Extension Apparatus in Fracture of the Thigh. By Gurdon S. Buck, M. D.... 356
Disease, Epidemic. Health of Buffalo... 33
Diseases, infectious, Contagious and Pestilential.... 441
Disease, Nature in.... 108
Diseases of the Throat and Air Passages. By Edward B. Stevens, M. D....50, 99, 181
Dissertation on some of the actions of Mercury. By J. R. Lothrop, M. D.... 369
Dixon, James, M. D., F. R. C. S. Diseases of the Eye.... 196
Dorland, Philip S., M. D., Death of.... 399
Dressing of new-born Infants.... 97
Drug Powdering.... 272
Dysmenorrhœa, Surgical Treatment of. By J. F. Miner, M. D.... 87

Page.

**E.**

Education, Medical.... 75
Emmet, Thomas A. Accidental and Congenital Atresia of the Vagina.... 583
Emmet, Thomas A. Reduction of Inverted Uteri by a new method.... 406
Epidemic Cholera, remarkable exemption of Buffalo from.... 150
Epidemic Disease, Health of Buffalo.... 33
Erie County Medical Society, Annual Meeting of.... [illegible] 320
Erie County Medical Society, Semi-Annual Meeting of....41, 450
Ether Spray in Strangulated Hernia.... 190
Every Saturday....280, 484
Examinations for Life Insurance.... 476
Exchange Journals.... 39
Existence of a Flourescent substance closely resembling Quinine in the Animal Texture.... 67
Extra Uterine Pregnancy. By S. F. Clark, M. D.... 135
Extract, Report of Deaths in Buffalo, 81, 410, 450
Extract, Report of Superintendent of the Buffalo General Hospital.... 231
Extract, Address read before the Indiana State Medical Society.... 400

**F.**

Facts connected with the recent spread of Cholera.... 54
Farre, Frederic John, M. D., F. L. S., Materia Medica and Therapeutics.... 195
Febris Nigra—Black Death.... 28
Fevers, Typhus and Typhoid.... 82
Fifield, W. C. B., M. D., Surgical Clinic of La Charite, by Prof. Velpeau. (Translated from the French).... 278
First appearance of Man on our Planet.... 368
Flint, Austin, M. D. Diseases of the Respiratory Organs.... 154
Flint, Austin, M. D. Practice of Medicine. 276
Flourescent substance closely resembling Quinine existing in the Texture of Animals.... 67
Foreign Bodies in the Nasal Passages. By M. H. Shaw, M. D.... 270
Fractures of the Radius at the Styloid Process, treatment of, by Gordon's Splints, 141
Fractures of the Thigh, Improved Extension Apparatus. By Gurden S. Buck.. 356
Fractures, Treatment of. By C. C. F. Gay, M. D.... 451
Francis, Samuel W., A. M., M. D. Biographical Sketches of New York Surgeons.... 39
Furman, Guido, M. D. Medical Register of the City of New York.... 79

**G.**

Gaillard, E. S., M. D. Diphtheria.... 447
Garrett, Alfred C., M. D. Guide for using Medical Batteries.... 482
Garrett, Alfred C., M. D. Medical Electricity.... 111
Gay, C. C. F., M. D., Letter from.... 13
Gay, C. C. F., M. D. Protoxyde of Azote as an Anaesthetic agent. (Translated from the French.... 43
Gay, C. C. F., M. D. Treatment of Fractures.... 451
Gleet, Insufflation of Medicated Powders, 107
Godey's Lady's Book.... 280
Gordon's Splint in the Treatment of Fracture of the Radius at the Styloid Process.... 141
Gould, William, M. D. Valedictory Address.... 411

### INDEX.

Page.

Greeley, Horace. The American Conflict .. 448
Gunning, Thomas Brain. Treatment of Fracture of the lower Jaw, by Interdental Splints .......... 407

### H.

Half-Yearly Abstract of Medical Sciences .. 446
Hamilton, Frank Hastings, B. A., A. M., M. D. Fractures and Dislocations ..... 153
Health of Buffalo—Epidemic Diseases ...... 33
Heart's Contraction, Nature of .......... 107
Hemoptysis, a certain form, unassociated with Pulmonary Tuberculosis .......... 69
Hernia, Case of Congenital Strangulated. By John Root, M. D .......... 474
History of Diphtheria in the town of Hamburg and vicinity. By George Abbott, M. D. .......... 241
Hodges, Richard M., M. D. Practical Dissections .......... 446
Hæmorrhagica Purpura and Hæmatomesis. By J. F. Norton, M. D .......... 268
Homœopathy in the University of Michigan 336
Hospital Elections .......... 235
Hydrocele treated by Ammonio Ferric Alum. By H. R. Rogers, M. D .......... 418
Hyposulphite of Soda in the treatment of Diphtheria .......... 73

### I.

Information for persons desirous of entering the Medical Corps of the Army ..... 74
Improved Extension Apparatus for the Treatment of Fractures of the Thigh. By Gurdon S. Buck, M. D. .......... 356
Infectious, Contagious and Pestilential Diseases .......... 441
Insufflation of Medicated Powders in Gleet, 107
Interesting Cases in Private Practice. By A. J. Scott, M. D. .......... 128
Internal use of Chloroform in the Treatment of Delirium Tremens .......... 145
International Medical Congress .......... 189
Irvin, Gailbraith, A., M. D., Death of ...... 400

### J.

Jansen, A., M. D. A case of Constipation with a subsequent Discharge of Living Maggots .......... 281
Jones, C. Handfield, M. B., F. R. C. S. Nervous Disorders .......... 238
Jones, Joseph, M. D. Researches upon Spurious Vaccination .......... 417
Journal of Psychological Medicine and Medical Jurisprudence .......... 483

### L.

Lake Erie Medical Society. Hydrocle treated by Ammonio Ferric Alum. By H. R. Rogers. M. D. .......... 418
Lawrence, John Z., F. R. C. S., M. D., and Robert C. Moon. Hand Book on Opthalmic Surgery .......... 155
Lecture on the Physiological and Remedial Effects of Increased Pressure of the Atmosphere. By Charles A. Lee, M. D... 199
Lecture upon Recent Observations on Medical Subjects in Europe. By James P. White .......... 159
Lee, Charles A., M. D., Letter from, with Statistics .......... 390
Lee, Charles A., M. D. Lecture on the Physiological and Remedial Effects of Compressed Air .......... 199
Lee, Charles A., M. D. Provision for the Insane Poor of the State of New York, 236

Page.

Lee, Benj., M. D. Contribution to the Pathology, Diagnosis and Treatment of Angular Curvatures .......... 363
Letter from George Abbott, M. D. .......... 302
Letter from C. C. F. Gay, M. D. .......... 13
Letter from Wm. M. Cornell, M. D. .......... 14
Letter from a Converted Allopathist ....... 15
Letter from J. F. Norton, M. D. .......... 268
Letter from M. H. Shaw, M. D. .......... 270
Letter from Northwestern Christian Advocate .......... 390
Letter from Charles A. Lee, M. D. .......... 390
Letter from C. H. S. Davis, M. D. .......... 396
Leucorrhœa, Iodine in the Treatment of ... 69
Life Insurance, Examinations for .......... 476
Local Anæsthesia .......... 30
Lothrop, J. R., M. D. Dissertation upon some of the actions of Mercury ....... 369
Lothrop, J. R., M. D. Verdict against an Apothecary for an alleged mistake in putting up a Prescription ..........85, 117
Lothrop, J. R., M. D. Verdict against an Apothecary for a mistake in putting up Veratrum Viride, instead of Valerian .. 343

### M.

Mackay, Edward, M. D., Death of .......... 478
Maggots, living. A case on Constipation with a subsequent discharge of. By A. Jansen, M. D .......... 281
Man, first appearance of, upon our Planet .. 368
Medical Association, American .......... 321
Medical Association, American, Eighteenth Annual Meeting of .......... 420
Medical Association, Buffalo, Proceedings of....49, 133, 179, 214, 281, 354, 369, 411, 451
Medical Convention .......... 320
Medical Education .......... 75
Medical International Congress .......... 189
Medical Society of Erie County, Annual Meeting of .......... 327
Medical Society of Erie County, Semi-Annual Meeting of ..........41, 450
Medical Society, Lake Erie—Hydrocle treated by Ammonio Ferric Alum. By H. R. Rogers, M. D. .......... 418
Medicated Powders, Insufflation of, in Gleet .......... 107
Medical Teachers' Convention .......... 404
Medicines Received .......... 192
Meigs, Charles D., M. D. Obstetrics—The Science and the Art .......... 445
Memorandum for the Information of Persons desirous of entering the Medical Corps of the Army .......... 74
Menstruation of a Child five years of age ... 66
Metallic Spectacles .......... 85
Meyler, J. J., M. D. Watson Abridged ... 482
Miner, J. F., M. D. Exsection of Fibula ... 265
Miner, J. F., M. D. Exsection of Hip-joint .......... 265
Miner, J. F., M. D. Orthopædic Surgery .. 9
Miner, J. F., M. D. Ovarian Tumor, Removal of, with fatal result .......... 45
Miner, J. F., M. D. Successful Removal of an Ovarian Tumor .......... 451
Miner, J. F., M. D. Surgical Treatment of Dysmenorrhœa .......... 87
Moon, Robert C., M. D., and John C. Lawrence, F. R. C. S., M. D. Hand-Book on Ophthalmic Surgery .......... 155
Moter Nerves, Termination of, in Muscles .. 104

### N.

Narciene .......... 442
Nasal Polypus. By Charles S. Davis, M. D., 259
Nature of Disease .......... 108
Nature of Heart's Contraction .......... 107

Page.

Nelson, R., M. D. Asiatic Cholera........ 38
Neligan, J. Moore, M. D., M. R. I. A. Diseases of the Skin.................... 154
Nerves, Moter, Termination of, in Muscles, 104
Nervous Ithiopathic Albumenuria and the Albumenuria of Diseased Kidneys, mode of distinguishing between............ 27
New Anæsthetic.......................... 103
New-Born Infants, Dressing of............ 97
New Form of Antiseptic for Local Use.... 442
New Instrument for Subcutaneous Injections..................................... 138
New Sydenham Society.................... 40
Norton, J. F., M. D. Purpura Hæmorrhagica and Hæmatomesis................ 268
North, Nelson L. A Theory of Inflammation....................................... 482
Notes, Clinical—Exsection of Fibula........ 265
Notes, Clinical—Exsection of Hip-joint.... 265
Notes on a case of Aphasia. By Joseph G. Richardson, M. D.................... 211

**O.**

Obituary—Phillip S. Dorland, M D........ 399
Obituary—Gailbraith A. Irvin, M. D....... 400
Obituary—Edward Mackay.................. 478
Ohio State Medical Transactions.......... 158
On a certain form of Hœmoptysis unassociated with Pulmonary Tuberculosis..... 69
On the mutual action of the Elements of Soluble Salts without and within the Animal Economy.......................... 139
On the Existence in the Texture of Animals of a Flourescent Substance closely resembling Quinine..................... 67
Orthopedic Surgery. By J. F. Miner, M. D., 9
Ovariotomy.................................. 368
Ovariotomy — Report of two successful cases. By James P. White, M. D.... 481
Ovariotomy—Report of a successful case of. By J. F. Miner, M. D.............. 481
Ovariatomy—Removal of a Multilocular Ovarian Tumor weighing nineteen and a half pounds, with fatal result. By J. F. Miner, M. D......................... 45

**P.**

Payne, W., M. D. Principles of Medicine.. 279
Percy, Samuel R., M. D. Digtialin......... 447
Percy. Samuel R., M. D. What Effect has the Meat or Milk from Diseased Animals upon the Public Health........... 446
Photographs of the Presidents and Acting Presidents of the American Medical Association.................................. 484
Physicians' Advertisement.................. 95
Physicians' Pocket Record.................. 194
Pilulae Metalorum et Amarum.............. 481
Pneumonia, Acute, Treatment of.......... 28
Poison, the Rinderpest.................... 66
Poisoning by Strychnia. By S. T. Clark, M. D.................................... 135
Polypus. Nasal. By Charles S. Davis, M. D.................................... 259
Poultices, Use and Abuse of................ 102
Practical Papers on Diseases of the Throat and Air Passages. By Edward B. Stevens, M. D..........................50. 99, 181
Practice of "Specialties" in Medicine..... 233
Pregnancy, Extra Uterine. By S. T. Clark, M. D.................................... 135
Profession, Quackery in.................... 188
Protoxyde of Azote as an Anæsthetic agent. (Translated from the French) By C. C. F. Gay, M. D.......................... 43
Probable Appearance, Cause and Prevention of Cholera......................... 443
Public Press and Criminal Abortion........ 358

Page.

Purpura Hæmorrhagica and Hæmatomesis. By J. F. Norton, M. D.................. 268
Pyæmia after Amputation. By E. R. Barnes, M. D.................................. 385

**Q.**

Quackery in the Profession................ 188

**R.**

Rand, B. Edward, M. D. Elements of Medical Chemistry........................... 155
Remarkable Exemption of Buffalo from Epidemic Cholera and the usual Diseases of the Season............................. 150
Removal of a large Bronchocele............ 33
Report of Cholera Conference at Constantinople..................................... 17
Resolutions of the Conventions of Medical Teachers................................. 404
Reviews—American Conflict. By Horace Greely,.......................... 444
A Theory of Inflammation. By Nelson L. North, M. D.......... 483
American Naturalist.............. 449
Asiatic Cholera. By F. S. Burrall, M. D.............................. 38
Asiatic Cholera. By R. Nelson, M. D.............................. 38
Asiatic Cholera, By E. Whitney, M. D. and N. B. Whitney, M. D. 199
Atresia of the Vagina. By Thos. A. Emmett, M. D............... 483
Biographical Sketches of New York Surgeons. By Samuel W. Francis, M. D., A. M........... 39
Beacon. By Wm. Cornell, M. D., L. L. D.......................... 79
Clinical Observations on Functional and Nervous Disorders. By C. Handfield Jones, M. B., F. R. C. P...................... 233
Clinical Lectures upon Amblyopia and Amaurosis and Extraction of Cataract. By Prof. A. Von Græfe. Translated from German, by Hasket Derby, M. D.. 238
Common Nature of Epidemics and their relation to Climate and Civilization. By Southwood Smith, M. D............. 279
Contributions to the Pathology, Diagnosis and Treatment of Angular Curvatures. By Benj. Lee, M. D..................... 362
Digitalin. By Samuel R. Percy, M. D............................ 447
Diphtheria. By R. S. Gaillard, M. D............................ 447
Diseases of the Respiratory organs. By Austin Flint, M. D.. 154
Diseases of the Skin. By J. Moore Neligan, M. D., M R. I A. 154
Diseases of the Digestive Organs, Functionally Treated. By Thos. King Chambers, M. D......... 405
Excision of the Superior Maxilla. By William B. Whitehead, M. D. 237
Fractures and Dislocations. By Frank Hastings Hamilton, A. B., A. M., M. D............... 153
Functions and Disorders of the Reproductive Organs, in Childhood, Youth, Adult Age and Advanced Life. By William Acton, M. R. C. S............... 319
Guide for the use of Medical Batteries. By Alfred C. Garrett, M. D............................ 482

Page.
Reviews—Half-Yearly Abstract of Medical Sciences........ 446
Infantile Paralysis and its attendant Deformities By Charles Fayette Taylor, M. D........ 279
Injuries of the Spine. By John Ashurst. A. M., M. D........ 263
Index of Diseases and their Treatment. By Thomas Hawks Tanner, M. D........ 406
Inhalations in the Treatment of Diseases of the Air Passages. By J. M. Da Costa, M. D........ 407
Luxations of Femur in Ischiatic Notch. By Lewis A. Sayre, M. D........ 448
Materia Medica and Therapeutics. By Frederic John Farre, M. D., F. L. S........ 195
Mechanical Treatment of Inflamed Joints. By Lewis A. Sayre, M. D........ 38
Medical Diagnosis. By J. M. Da Costa, M. D........ 110
Medical Electricity. By Alfred C. Garrett, M. D........ 111
Medical Chemistry. By B. Edward Rand, M. D........ 115
Medical Jurisprudence. By Alfred Swain Taylor, M. D., F. R. S........ 236
Medical Register of the City of New York. By Guido Furman, M. D........ 79
Medical Register of the District of Columbia. By J. M. Toner, M. D........ 363
Modern Anesthesia. By Truman Smith, M. C........ 448
Nitrous Oxide. By George F. Baker, D. D. S........ 112
Notes on Epidemics for the Public. By Francis Edmund Anstie, M D., F. R. C. P........ 239
Obstetrics—the Science and the Art. By Charles D. Meigs, M. D........ 445
Œsophagotomy for the Removal of Foreign Bodies. By David W. Cheever, M. D........ 405
Opthalmic Science. By Henry W. Williams, M. D........ 37
Ophthalmic Surgery. By John Z. Lawrence. F. R. C. S., M. D., and Robert C. Moore, M. D........ 155
Practical Dissections. By Rich'd D. Hodges, M. D........ 446
Practical Chemistry. By John C. Bowman, M. D., F. C. S........ 239
Practice of Medicine. By Austin Flint, M D........ 275
Practical Study of Diseases of the Eye. By James Dixon, M. D., F. R. C. S........ 196
Practical Therapeutics. By Edward John Waring, F. R. C. S., F. L. S........ 277
Principles and Practice of Medicine, and Pathology. By W. Payne, M. D........ 279
Principles of Surgery based on Pathology. By Wm. Canniff, M. D., M. R. C. S........ 194
Provision for the Insane Poor of the State of New York. By Charles A. Lee, M. D........ 236
Reduction of Inverted Uteri, by a new Method. By Thomas A. Emmet, M. D........ 405

Page
Reviews—Report to the War Department upon Prevailing Diseases of the United States. By J. H. Baxter, M. D., Colonel U. S. Vols........ 237
Researches upon Spurious Vaccination. By Joseph Jones, M. D. 447
Renewal of Life. By Thomas King Chambers, M. D........ 318
Science and Practice of Medicine. By William Aitkin, M. D. Vol. 1 238
Spermatorrhœa. By Robert Bartholow, M. D........ 79
Surgical Clinic of La Charite. By Professor Velpeau. Translated by W. B. Fifield, M. D........ 278
The Brain and Cranial Nerves. By Thomas S. Bulmer........ 197
Transactions Indiana State Medical Society........ 111
Transactions Ohio State Medical Society........ 158
Transactions Pennsylvania State Medical Society........ 237
Transactions Vermont State Medical Society........ 239
Treatment of Fracture of Lower Jaw by Interdental Splints. By Thomas Brain Gunning........ 406
Urinary and Renal Disease. By William Roberts, M. D........ 78
Uterine Surgery. By J. Marion Sims, A. B., M. D........ 36
Vesico, Vaginal Fistula. By M. Shuppert, M. D........ 239
Watson Abridged. By J. J. Meyler, A M. M. D........ 482
What effect has the Meat or Milk from Diseased Animals on the Public Health. By Samuel R. Percy, M. D........ 446
Report to the International Sanitary Conference. Translated by Samuel L. Abbott, M. D........ 482
Rheumatism, Acute, Trousseau's Syrup of Lime in........ 440
Richardson, Joseph G Notes ona case of Aphasia with some Suggestions towards a new Hypothesis of Paralysis........ 211
Rinderpest Poison........ 66
Roberts, William, M. D. A Practical Treatise on Urinary and Renal Diseases........ 78
Rochester, Thomas F., M. D. Remarks on Cholera, (continued)........ 1
Rogers, H. R., M. D. Hydrocle treated by Ammonio Ferric Alum........ 418
Root, John, M. D. Case of Congenitat Strangulated Hernia........ 474

S.

Sanitary Commission, Contributions for the Medical Volume........ 106
Sayre, Lewis A., M. D. Luxation of Femur into Ischiatic Notch........ 448
Sayre, Lewis A., M. D. Mechanical Treatment of Inflamed Joints........ 38
Schuppert, M., M. D. Vesico Vaginal Fistula........ 239
Scott, A. J., M. D. Interesting Case from Private Practice........ 128
Shaw, M. H., M. D. Foreign Bodies in Nasal Passages........ 270
Sims, J. Marion, A. B., M. D. Uterine Surgery........ 36
Smith, Southwood, M D. Common Nature of Epidemics and their relation to Civilization........ 279

Page.
Smith, Truman, M. C. Modern Anæsthesia 448
Society, Erie County Medical, Meeting of 320
Society, Erie County Medical, Smi-Annual Meeting of 41, 450
Society, New Sydenham 40
Soda, Citrate of, in Diabetes 106
"Specialities," Practice of, in Medicine 233
State Medical Society, Annual Meeting of 235
Stevens, Edward B., M. D. Practical Papers on Diseases of the Throat and Air Passages 50, 99, 181
Strangulated Hernia, Ether Spray in 190
Strychnia, Case of Poisoning By S. T. Clark, M. D. 135
Subcutaneous Injection, New Instrument for 138
"Surgical Remedies," Vesicants, Rubifacents, Setons, Issues, etc. 281

**T.**

Tanner, Thomas Hawks, M. D. Index of Diseases and their Treatment 406
Tayler, Alfred Swain, M. D., F. R. S. Medical Jurisprudence 236
Taylor, Charles Fayette, Infantile Paralysis and its attendant Deformities 279
Termination of the Moter Nerves in Muscles 104
The Head-Quarters of Drunkenness 185
The Nation 280, 484
Toner, J. M., M. D. Medical Register of the District of Columbia 363
Treatment of Pneumonia 28
Treatment of Cholera by Hypodemic Injections of Warm Water 143
Treatment of Consumption by Hygiene, Climate and Medicine. By Henry Bennet, M. D. 217, 307
Treatment of Diphtheria with Hyposulphite of Soda 73
Treatment of Fractures, By C. C. F. Gray, M. D. 451
Treatment Fractures of the Radius at the Styloid Process by Gorden's Splints 141

Page.
Trousseau's Syrup of Lime in Acute Rheumatism 440

**U.**

University of Michigan, Homœopathy in 366
Urachus Pervious, after Birth 106
Use and Abuse of Poultices 102
Uterus, forcible tearing away of 32

**V.**

Valedictory Address. By William Gould, M. D. 411
Velpeau, Professor. Surgical Clinic at La Charite. Translated by W. C. B. Fifield, M. D. 278
Verdict against an Apothecary for an alleged mistake in putting up a prescription. By J. R. Lothrop, M. D. 85, 117
Verdict against an Apothecary for a mistake in putting up Veratrnm Viride instead of Valeriam. By J. R. Lothrop, M. D. 343
Vivisections 152
Volume vi, Completion of 478
Von Graefe Prof., on Amplyopia and Amaurosis and Extraction of Cataract. Translated by Hasket Derby, M. D. 238

**W.**

Warning, Edward John. F. R. C. S., F. L. S. Practical Therapeutic 277
White, James P., M. D. Return of 82
White, James P., M. D. Charge to the Graduating Class 326
White, James P., M. D. Lecture upon Recent Observations upon Medical Subjects in Europe 159
White, James P., M. D. Report of two successful Cases of Overiatomy 451
Whitehead, Wm. B., M. D., on Excision of the Superior Maxilla 237
Whitney, E., M. D., and Whitney, E. B., M. D. Asiatic Cholera 196
Williams, Henry W., M. D. Recent Advances in Ophthalmic Science 37

### Books and Pamphlets Received.

On Spermatorrhœa; its Causes, Symptomatology, Pathology, Prognosis, Diagnosis, and Treatment. By Roberts Bartholow, A. M., M. D., Professor of Physics and Medical Chemistry in the Medical College of Ohio; Lecturer on Clinical Medicine, and Physician to St. John's Hospital, Cincinnati, Assistant Surgeon U. S. Army, etc., etc. New York: Wm. Wood & Co., 51 Walker street, 1866.

The Medical Register of the City of New York, for the year commencing June 1, 1866; published under the supervision of the "New York Medico-Historical Society." Guido Furman, editor. For sale by Wm. Wood & Co., 61 Walker street.

The Key to General Practical Ventilation found; the ready means, awaiting a proper use, exemplified through this study, and the production of the "Impinged Draught,"—"Wind-Guard." A Chimney-top aud Ventilator, in cast iron and wrought metal, as required.

Twenty-first Annual Announcement of the Ohio College of Dental Surgery, College street, Cincinnati, O. Second edition.

---

The following Journals are regularly received in exchange:—London Lancet—Editors, J. H. Bennett, M. D., T. Wakely, jr., M. R. C. S. E.; Braithwaite's Retrnspect of Practical Medicine and Surgery. New York: W. A. Townsend, 434 Broome street; The Ophthalmic Review, edited by J. Z. Lawrence and Thomas Winslow, London; Canada Medical Journal, edited by G. E. Fenwick, M. D. and F. W. Campbell, M. D., L. R. C. P. L.; American Journal of the Medical Sciences, edited by Isaac Hays, M. D.; Boston Medical and Surgical Journal, edited by Samuel L. Abbott, M. D. and James C. White, M. D.; The American Journal of Insanity, edited by the officers of the New York State Lunatic Asylum; The New York Medical Journal; The Cincinnati Lancet and Observer, edited by Edward B. Stevens, M. D. and John A. Murphy, M. D.; The St. Louis Medical and Surgical Journal, edited by M. L. Linton, M. D. and Frank W. White, M. D.; The Medical Record; The Chicago Medical Journal; The Chicago Medical Examiner, edited by N. S. Davis, M. D.; The Medical and Surgical Reporter, edited by S. W. Butler, M. D.; The Cincinnati Journal of Medicine, edited by George C. Blackman, M. D., Theophilus Parvin, M. D. and Roberts Bartholow, M. D.; The

Richmond Medical Journal, edited by E. S. Gaillard, M. D. and W. S. McChesney, M. D.; The Savannah Journal of Medicine, edited by Juria Harris, M. D., J. B. Read, M. D. and J. G. Thomas, M. D.; The Medical Reporter, edited by J. S. B. Alleyne, M. D. and O. F. Potter, M. D.; The Detreit Review of Medicine and Pharmacy, edited by George T. Andrews, M. D., S. T. Duffield, M. D. and E. W. Jenks, M. D.; Atlantic Medical and Surgical Journal, edited by J. G. Westmoreland, M. D.; The Medical and Surgical Monthly, Memphis, Tenn., edited by Frank A. Ramsay, M. D., D. D. Saunders, M. D., E. Mills Willett, M. D. and William H. White, M. D.; Southern Journal of Medical Sciences, edited by E, D. Turner, M. D., D. Warren Brickell, M. D. and C. Beard, M. D., The Pacific Medical and Surgical Journal end Press, edited by Henry Gibbons, M. D.; The Galveston Medical Journal, edited by Greenvill Dowell, M. D.; The New Orleans Medical Record, a semi-monthly Journal of the Medical Sciances, edited by Bennett Dowler, M. D. and S. R. Chambers, M, D.; The American Journal of Pharmacy, edited by William Proctor, jr.; Medical News and Library, by Henry C. Lea, Philadelphia; The Druggist's Circular and Chemical Gazette; The Journal of Materia Medica, by Joseph Bates, M. D. and H. A. Tilden; The Dental Cosmos, edited by J. H. McQuillen, D. D. S. and George J. Zeigler, M. D.; The Atlantic Monthly, published by Ticknor, Fields & Co., Boston; Godey's Lady's Book, published by Louis A. Godey, Philadelphia; The Herald of Health; Eclectic Medical and Surgical Journal, Philadelphia; Dental Times, Philadelphia; Eclectic Medical Journal, Cincinnati; American Eclectio Medical Review, New York; Eclectic Medical Journal, Cincinnati, Ohio.

